# Supplementary figures and images for: Biological effects of carbon nanotubes generated in forest wildfire ecosystems rich in resinous trees on native plants
Source: PeerJ. 2017 Aug 15;5:e3658. doi: 10.7717/peerj.3658 (PMC5562139; doi:10.7717/peerj.3658)

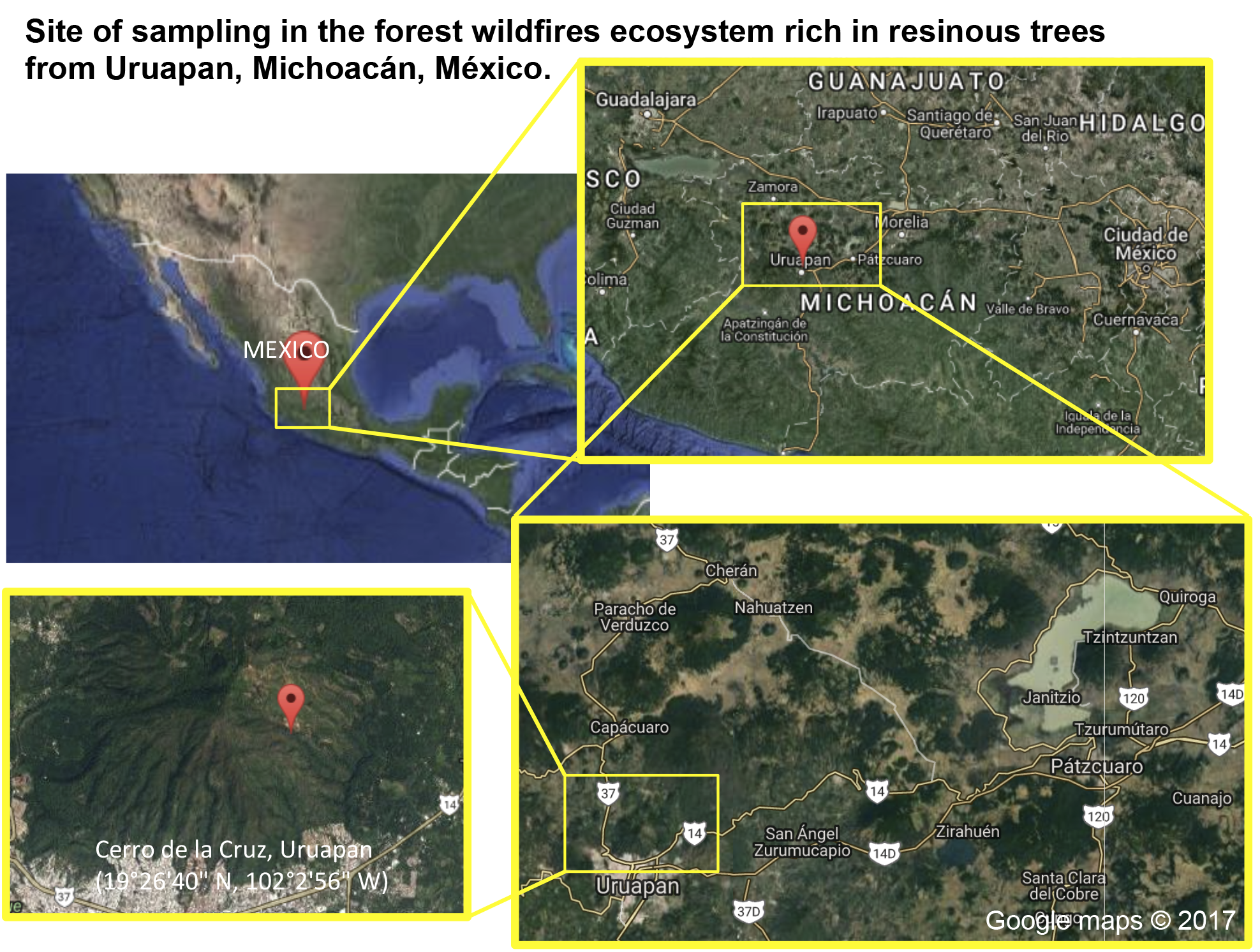

Supplement: File S1 [file peerj-05-3658-s001.png]

## Slide 1
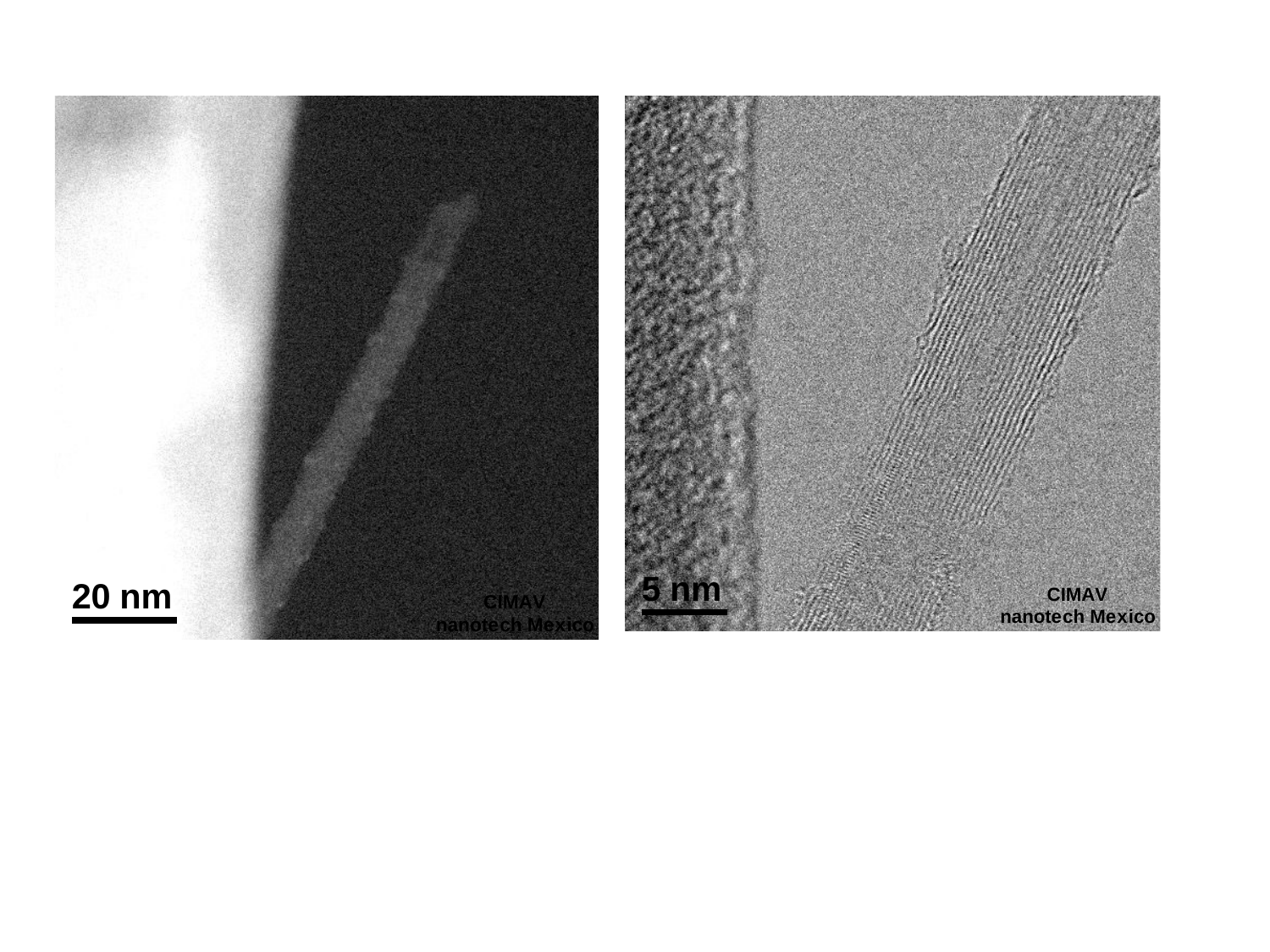

## Slide 2
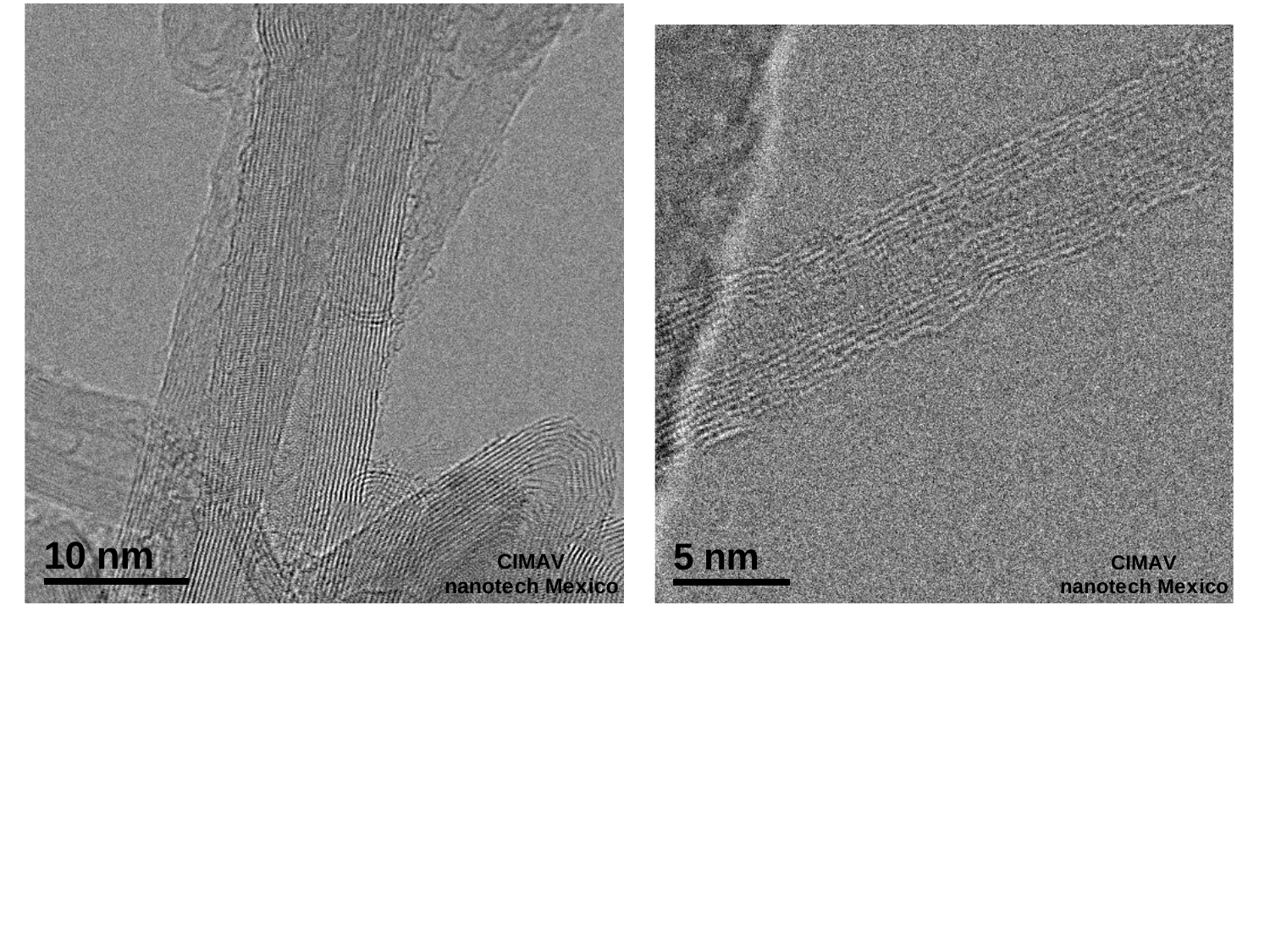

## Slide 3
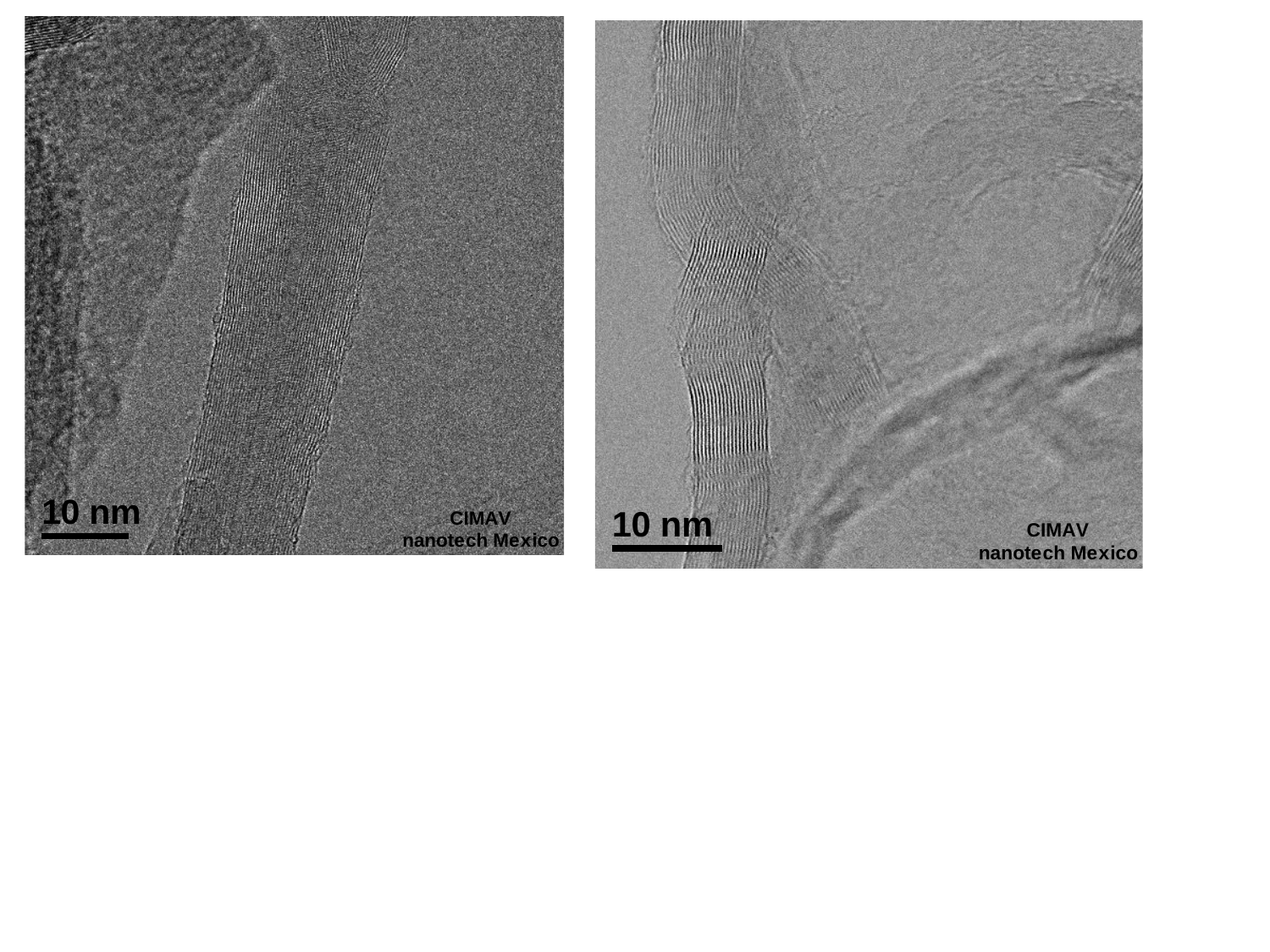

## Slide 4
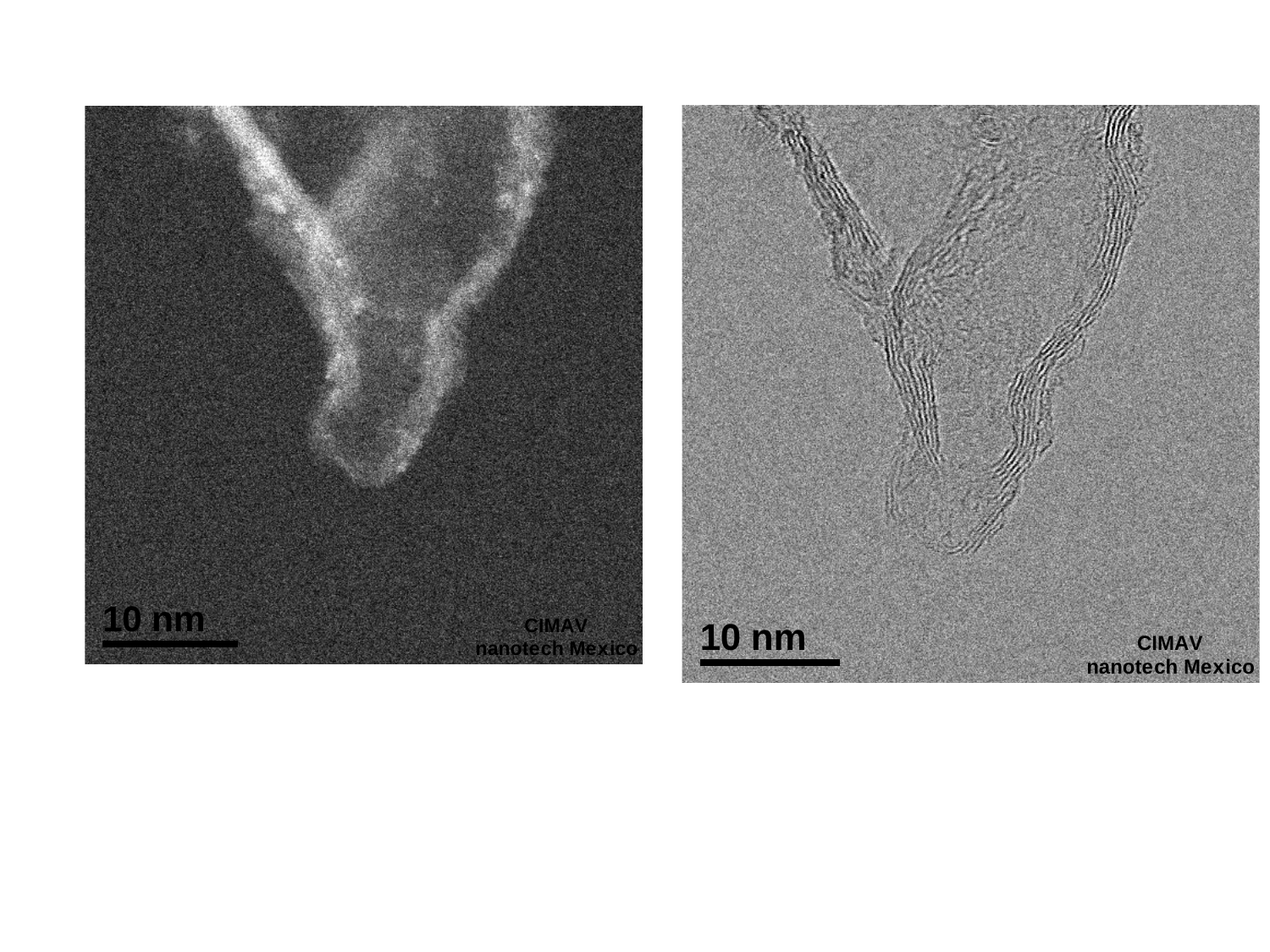

## Slide 5
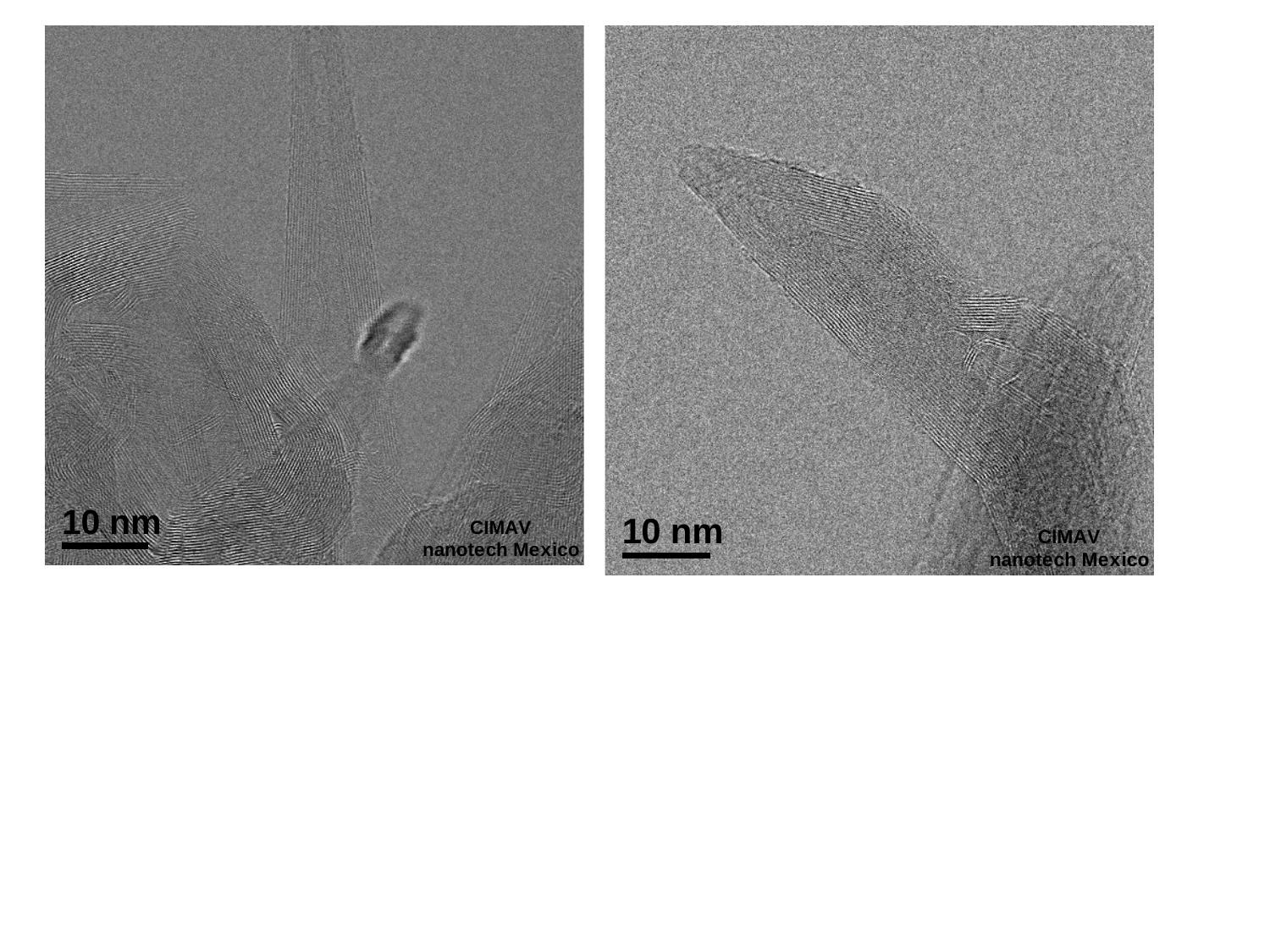

## Slide 6
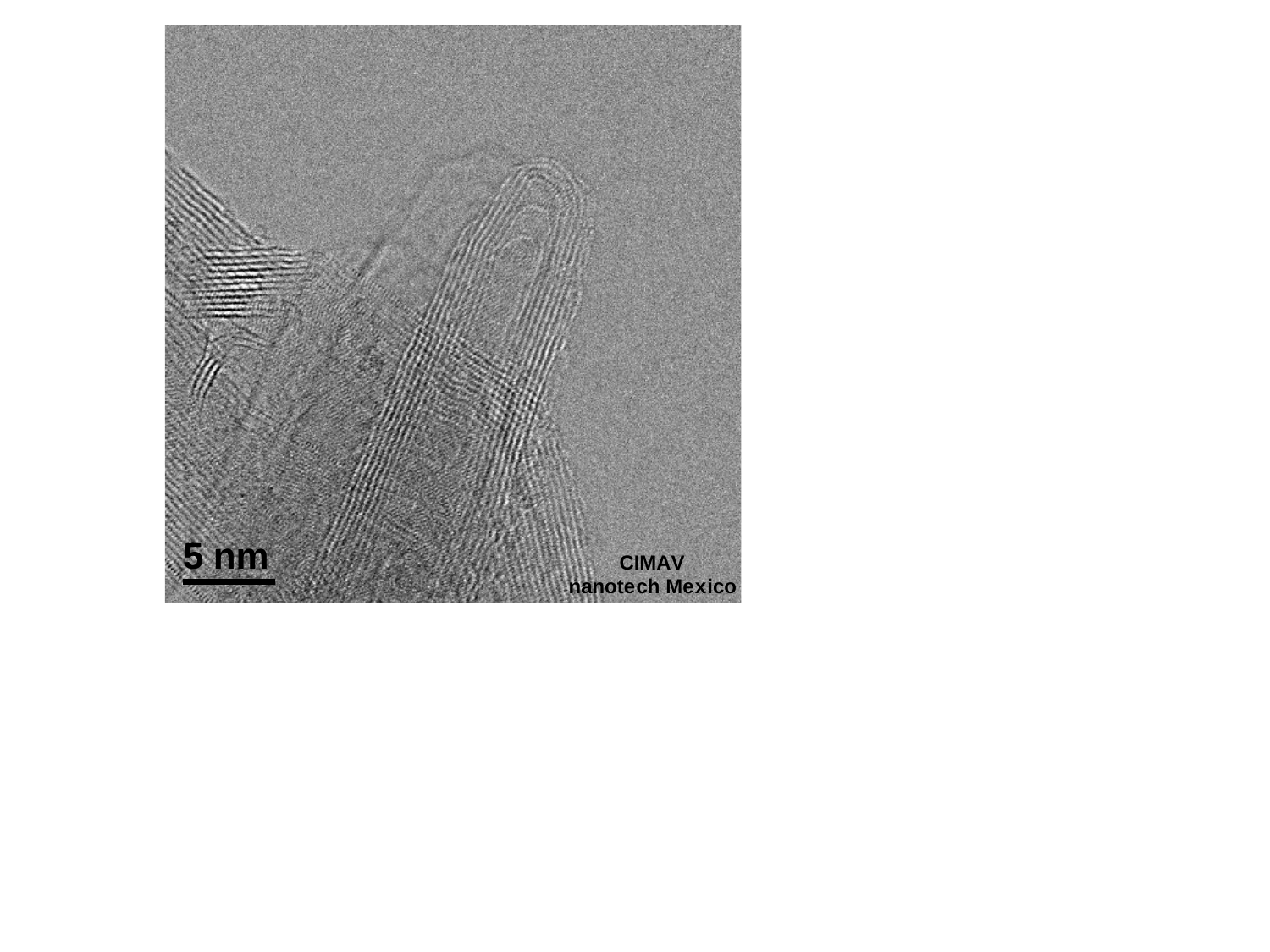

## Slide 7
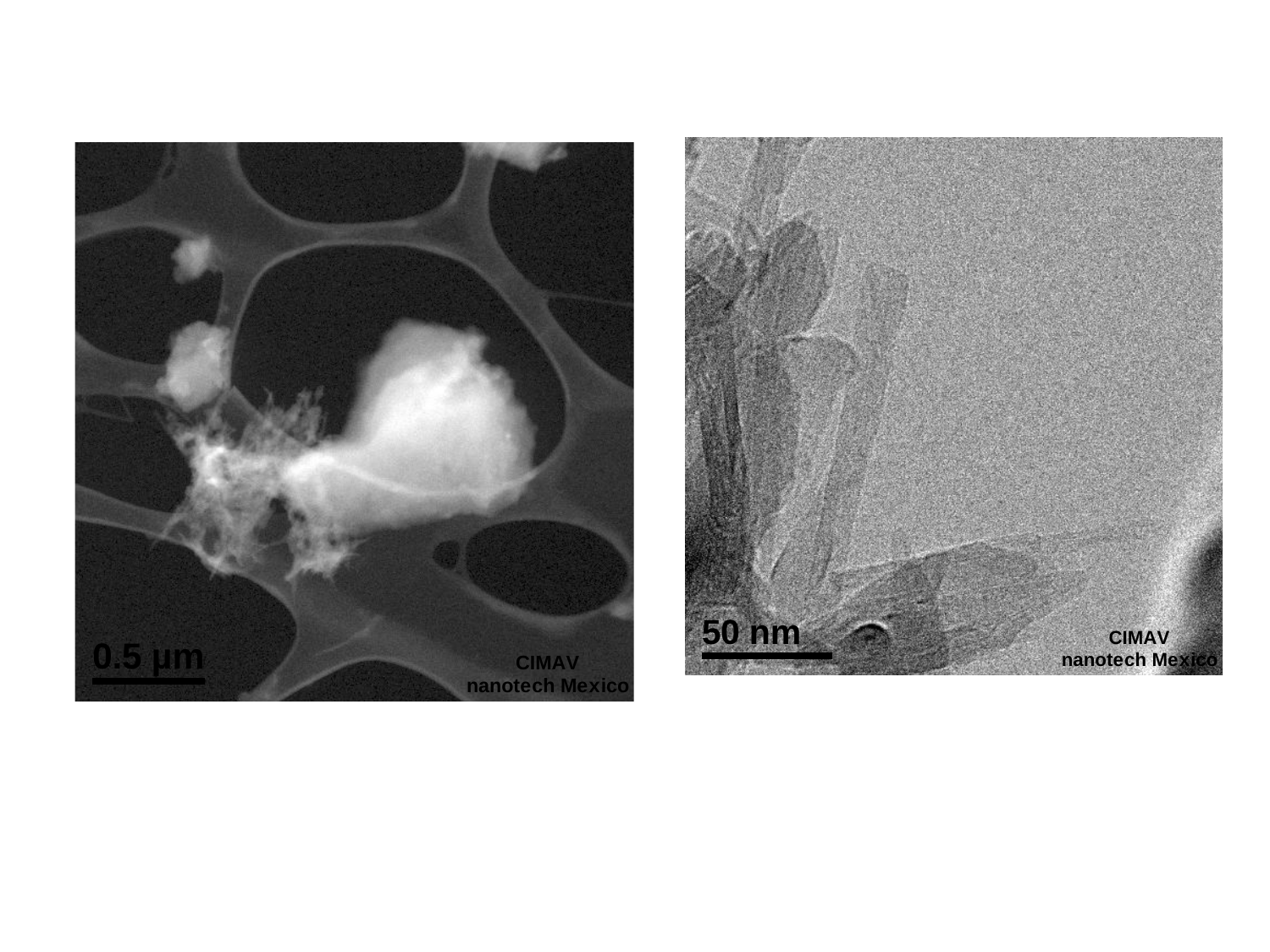

## Slide 8
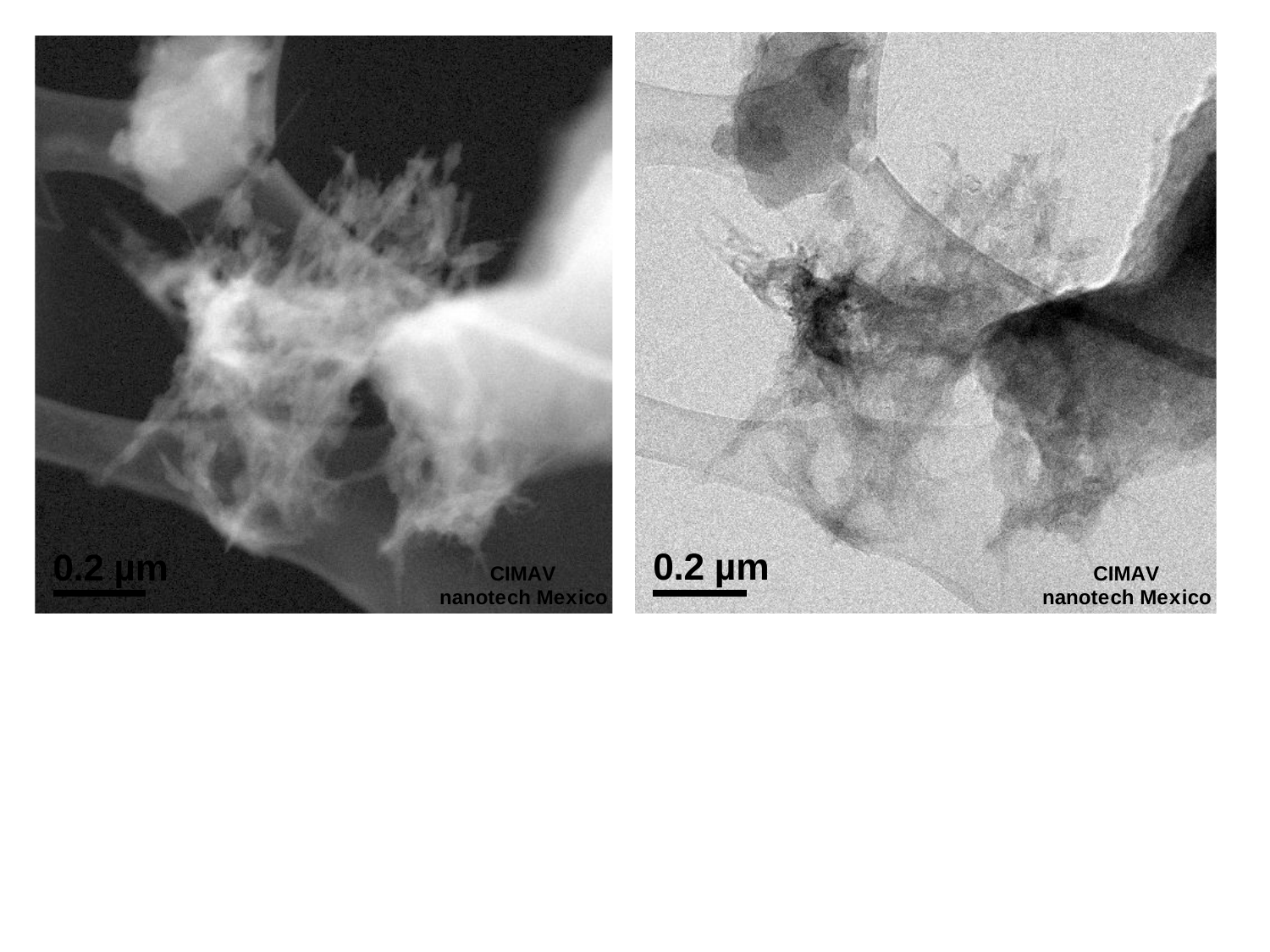

Supplement: Data S1 [file peerj-05-3658-s002.zip › rawdata/Pinus oocarpa - imagenes material suplementario (1 dia)[4].pptx]
